# Cell-Surface Signatures and Targets of Modulated Vascular Smooth Muscle Cells in Atherosclerosis: From State Identification to Precision Intervention

**DOI:** 10.3390/jcdd13090472

**Published:** 2026-09-20

**Authors:** Yinfei Xu, Yi Wang, Hui Wu, Pengfei Li, Jiaqi Zhuang, Zhean Shen, Ziyi Lu, Cheng Zhang, Yan Chen

**Affiliations:** 1 Department of Emergency and Critical Care Medicine, Suzhou Municipal Hospital, Gusu School, The Affiliated Suzhou Hospital of Nanjing Medical University, Nanjing Medical University, Suzhou 215000, China; chelsie.xu@stu.njmu.edu.cn (Y.X.); wangyi1@stu.njmu.edu.cn (Y.W.); wuhui9655@stu.njmu.edu.cn (H.W.); pengfeili@stu.njmu.edu.cn (P.L.); zheanshen@stu.njmu.edu.cn (Z.S.); 2 Department of Emergency and Critical Care Medicine, The First Affiliated Hospital of Nanjing Medical University, Nanjing 210029, China; zhuangjiaqi550@stu.njmu.edu.cn (J.Z.); belinda.lu@stu.njmu.edu.cn (Z.L.); 3 Basic Medical Research Center, The First Affiliated Hospital of Nanjing Medical University, Nanjing 210029, China; czhang@njmu.edu.cn; 4 Department of Emergency Management, School of Health Policy & Management, Nanjing Medical University, Nanjing 211166, China

**Keywords:** vascular smooth muscle cell, atherosclerosis, cell surface marker, phenotypic modulation, CITE-seq, targeted delivery, plaque stability, FAP, CD200, CD47

## Abstract

Atherosclerotic plaques contain vascular smooth muscle cell (VSMC)-derived populations that no longer fit a simple contractile-versus-synthetic model. Lineage tracing, single-cell transcriptomics, spatial profiling and multimodal surface-protein measurements now show that VSMC-derived cells occupy heterogeneous lesional states with pathogenic, reversible or plaque-stabilizing properties. This diversity creates a translational bottleneck. Intracellular markers and transcriptomic clusters can define state transitions, but they do not by themselves provide handles for live-cell isolation, molecular imaging, targeted delivery or selective intervention. This narrative review examines how VSMC state discovery can be translated into cell-surface signatures and surface-accessible intervention interfaces. We distinguish state/lineage markers, surface identification and sorting signatures, functional surface interfaces, causally supported candidate targets, and intervention-supported surface targets. Current evidence positions CD29, CD90, CD142, and CD200 primarily as tools for live-cell identification, whereas fibroblast activation protein (FAP) represents the most advanced example of an intervention-supported surface target for the depletion of a disease-associated modulated VSMC state. We propose a state-matched framework in which pathogenic states are selectively depleted, plastic or reversible states are modulated or reprogrammed, and matrix-supportive plaque-stabilizing states are preserved. C-C chemokine receptor type 2 (CCR2), guanylyl cyclase-B/natriuretic peptide receptor 2 (GC-B/NPR2), matrix metalloproteinase 14 (S14), and CD47 span different intermediate levels of therapeutic evidence, from targeted delivery to functional surface modulation and causal intervention, whereas CD36, triggering receptor expressed on myeloid cells 2 (TREM2), and integrins remain constrained by incomplete cell-state or lineage specificity. Major barriers include human protein-level and surface validation, state specificity, spatial accessibility and direct therapeutic testing.

## 1. Introduction

Atherosclerotic plaques are shaped not only by lipid deposition and immune inflammation but also by the capacity of vascular smooth muscle cells (VSMCs) to change state [1,2,3]. Classical pathology emphasized the migration of medial VSMCs into the intima, proliferation, extracellular matrix production and fibrous cap formation [4]. Later work reframed this process as phenotypic switching, in which contractile markers decline and synthetic, osteogenic, inflammatory or foam-cell-like features emerge [5,6]. More recent lineage-tracing and single-cell studies have sharpened the issue: lesional VSMC-derived cells are heterogeneous, and their contribution to plaque fate depends on which state they enter, where they are located and whether they can return to a stabilizing program [7,8,9,10].

This state heterogeneity matters because modulated VSMCs are not intrinsically harmful. Some VSMC-derived cells contribute collagen and extracellular matrix that help maintain fibrous cap integrity [8,11]. Others acquire lipid-loaded, inflammatory, matrix-degrading or senescent features that may enlarge the necrotic core, amplify inflammation or weaken plaque structure [7,12]. A therapeutic strategy that treats all modulated VSMCs as targets for removal would therefore risk eliminating cells that contribute to plaque stability. The central problem is not whether VSMCs switch phenotype but whether specific VSMC states can be identified, accessed and manipulated without damaging protective VSMC-derived populations.

Single-cell RNA sequencing has transformed the ability to define VSMC states. It can resolve contractile, fibromyocyte-like, chondromyocyte-like, fibroblast-like, proliferative and foam-cell-like clusters, and it can reconstruct transcriptional trajectories from contractile to modulated states [8,9,13]. However, transcriptional state definitions do not automatically solve translational access. A state can be recognized by RNA yet lack a validated protein marker. A protein can be expressed yet remain intracellular or non-specific [10]. A cell-surface marker can enable sorting yet have no causal role in disease [10]. A functional receptor can regulate state yet be shared by immune cells or endothelial cells [14,15]. These distinctions are not semantic. They determine whether a molecule should be described as a state/lineage identification marker, a surface identification/sorting signature, a functional surface interface, a causally supported candidate target, or an intervention-supported surface target.

This review focuses on that translational interface. It is not intended as another general review of VSMC phenotypic switching. Instead, it asks how transcriptionally and spatially defined VSMC states can be converted into surface-accessible identities that support live-cell identification, imaging, targeted delivery and selective intervention. There is an organizing axis: VSMC state heterogeneity → state identification → cell-surface signature → live-cell accessibility → therapeutic actionability → selective intervention → plaque fate. Throughout the review, we apply a translational evidence framework that distinguishes several complementary dimensions, including molecular localization, cellular/state specificity, mechanistic causality, human lesion validation, and therapeutic intervention. These dimensions are complementary rather than strictly ordinal: RNA expression does not establish protein expression or cell-surface localization; surface localization does not establish state specificity; human lesion association does not establish causality; and systemic intervention does not necessarily establish a VSMC-intrinsic mechanism.

**Figure 1:**
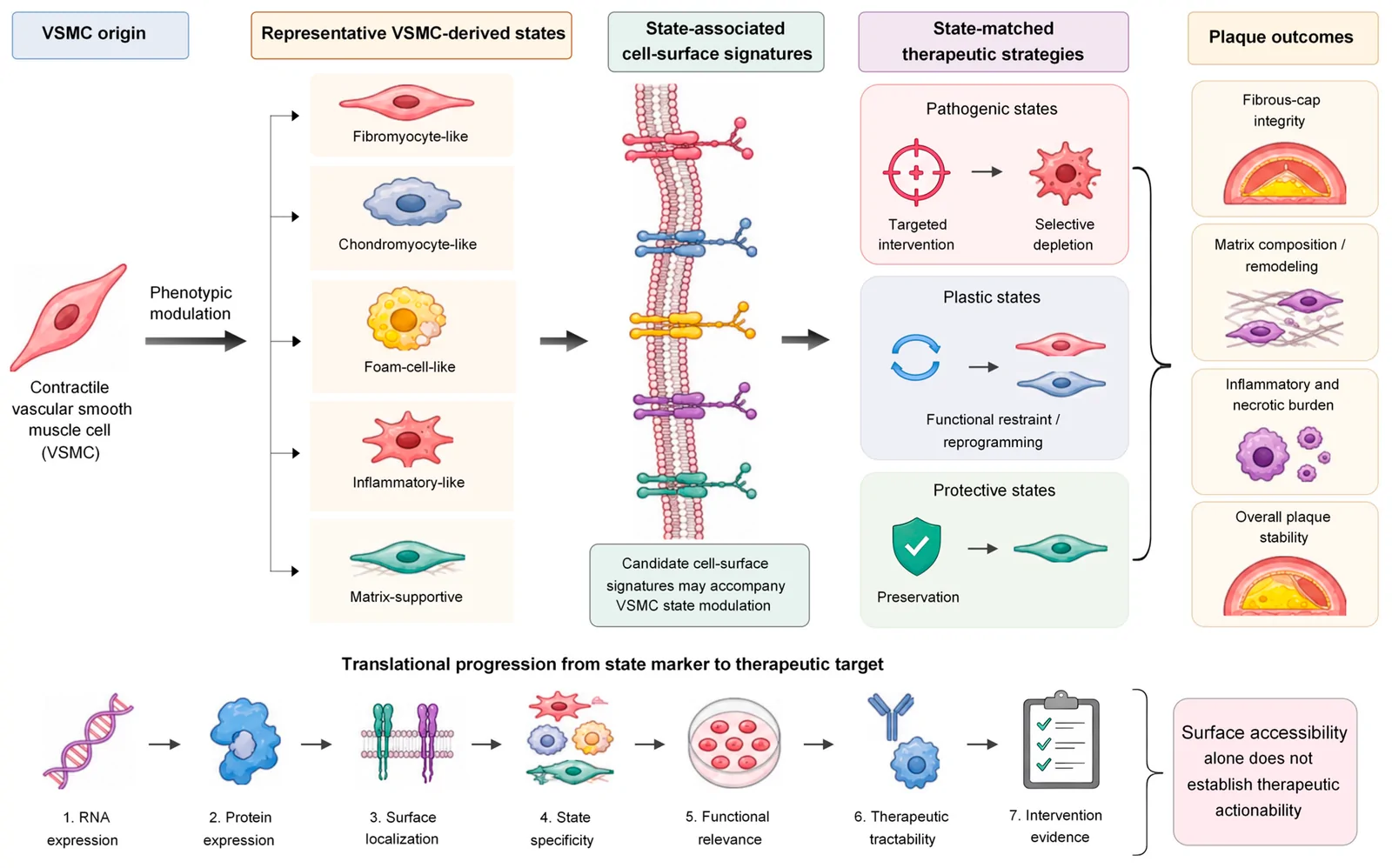
From VSMC states to surface-guided precision targeting. Arrows indicate conceptual relationships; colors distinguish different VSMC states and therapeutic strategies.

Pathogenic VSMC states with evidence of promoting plaque inflammation, foam-cell burden or matrix destabilization may be candidates for selective depletion [16,17,18]. Plastic or reversible states may be better suited to modulation or reprogramming [19]. Protective, matrix-producing or plaque-stabilizing states should be preserved [20]. This deplete-modulate/reprogram-preserve logic provides a more precise alternative to the simple idea that VSMC modulation should be blocked or that modulated VSMCs should be eliminated. The overall translational framework from VSMC state heterogeneity to surface-guided precision intervention is summarized in Figure 1.

### Literature Search and Evidence Selection

This narrative review was informed by a focused literature search of PubMed and Web of Science covering studies available from database inception through August 2026. Searches used combinations of terms related to vascular smooth muscle cells, atherosclerosis, phenotypic modulation, lineage tracing, single-cell and multimodal profiling, cell-surface markers, targeted delivery, and the individual candidate molecules discussed in this review. Recent mechanistic, single-cell, spatial, and intervention studies were prioritized, while earlier landmark studies were retained where necessary to provide conceptual context. Reference lists of key primary studies and recent reviews were also screened to identify additional relevant reports.

Priority was given to peer-reviewed original studies providing lineage-resolved evidence, human plaque data, multimodal transcript–protein profiling, direct surface or spatial validation, cell-specific genetic evidence, or in vivo therapeutic intervention. Reviews were used primarily for contextualization and citation tracking, whereas preprints were not used as the sole basis for target-level classification. Studies were considered for inclusion when they directly informed VSMC state identification, lineage attribution, surface accessibility, functional regulation, or therapeutic intervention in atherosclerosis. Studies without clear relevance to VSMC biology or atherosclerosis were excluded, whereas studies with insufficient evidence to support a specific target-level claim were not used as the basis for that classification. Because this is a narrative rather than a systematic review, study selection was guided by relevance to the predefined translational framework rather than by a formal systematic-review protocol.

## 2. Conceptual Basis: From VSMC State Heterogeneity to Surface Accessibility

### 2.1. VSMC State Heterogeneity and Loss of Traditional Markers

Traditional VSMC identity has often been defined by markers such as ACTA2, MYH11, transgelin (TAGLN) and calponin 1 (CNN1) [21]. These markers remain useful for contractile identity, but they become less reliable once VSMCs enter diseased lesions [22]. Lineage-tracing studies have shown that many VSMC-derived plaque cells downregulate canonical smooth muscle genes and adopt features that overlap with fibroblasts, chondrocytes, macrophages or foam cells [23,24]. This loss of marker fidelity creates a persistent interpretation problem. If a cell no longer expresses MYH11 or ACTA2, it may be mistaken for a non-VSMC lineage unless its ancestry is tracked [25].

Lineage tracing has therefore become essential for understanding VSMC contribution to plaques [7,26,27]. It has revealed that VSMC-derived cells contribute substantially to lesional cell populations that would be misclassified by canonical markers alone [28,29]. At the same time, lineage tracing has also exposed disagreement. Estimates of VSMC-to-macrophage-like transition vary depending on reporter system, digestion strategy, autofluorescence control, fluorescence-activated cell sorting (FACS) gating and transcript thresholding [4,8,30,31]. Multimodal profiling studies that combine lineage reporters, transcriptomes and surface proteins suggest that bona fide VSMC-derived macrophage-like cells may be less frequent than some earlier interpretations implied, while VSMC-derived fibromyocyte-like, chondromyocyte-like and foam-cell-like states remain robust features of advanced disease [32,33].

The implication is that intracellular or lineage markers define origin and state, but they do not by themselves provide a therapeutic handle. ACTA2 and MYH11 can help identify contractile programs [34,35]; Kruppel-like factor 4 (KLF4) can mark a regulatory transition [36], and lineage reporters can establish ancestry in mice. None of these is naturally suited for live-cell surface sorting or targeted delivery in human disease [37]. A translation-oriented VSMC review must therefore move from “what state is present?” to “which state can be accessed at the cell surface?”

A second implication is that state names should not be treated as fixed cell types [38]. A fibromyocyte-like cell, a chondromyocyte-like cell and a foam-cell-like state associated with VSMC lineage are operational states defined by a combination of lineage, transcriptome, protein expression, spatial location and disease context [20]. These states may overlap, interconvert or represent different points along a trajectory rather than terminal identities [39,40]. A summary of representative disease-associated VSMC states across studies, including their species, vascular beds, evidence basis, defining markers, spatial distribution, and inferred biological functions, is provided in Table 1. This context is essential because surface targeting requires more than simply naming a cluster. The target must be present on the relevant state at the relevant disease stage, remain accessible in intact tissue, and distinguish the intended state from neighboring protective or unrelated cells [41]. Without these filters, a state marker may become a misleading therapeutic label.

### 2.2. Why the Cell Surface Is the Translational Interface

VSMC phenotypic switching is governed by an interconnected regulatory network involving extracellular matrix remodeling, growth factor signaling, inflammatory pathways, and metabolic regulation [46,47]. Among these mechanisms, extracellular matrix remodeling represents a critical component of VSMC phenotypic modulation. Matrix metalloproteinases (MMPs), particularly MMP2 and MMP9, have been implicated in extracellular matrix turnover, VSMC migration, and the transition from a contractile phenotype toward synthetic, secretory, and matrix-remodeling states during vascular injury and atherosclerosis [48,49,50,51]. By modifying the extracellular environment and cell–matrix interactions, MMP activity contributes to the generation of disease-associated VSMC phenotypes [51]. Transforming growth factor-β1 (TGF-β1) represents another major regulatory axis of VSMC plasticity, integrating extracellular signals with SMAD-dependent and non-SMAD pathways to regulate contractile marker expression, extracellular matrix production, and fibroblast-like transition [52,53]. In addition, reduced vitamin D signaling has been associated with vascular remodeling and VSMC dysfunction, potentially affecting proliferation, inflammation, extracellular matrix homeostasis, and phenotypic stability [54,55]. Together, these regulatory mechanisms contribute to heterogeneous VSMC states and highlight the need for surface-accessible markers to identify and selectively target disease-relevant populations.

The cellular lipid bilayer membrane is the point at which cell identity becomes accessible. Cell-surface molecules can be recognized by antibodies, ligands, peptides, nanoparticles, engineered immune effectors and imaging tracers [56,57]. They can support fluorescence-activated cell sorting, mass cytometry, cellular indexing of transcriptomes and epitopes by sequencing (CITE-seq), in situ imaging, targeted RNA delivery or receptor modulation [58]. This accessibility explains why surface biology is a key bridge between single-cell discovery and clinical translation.

However, surface accessibility has several layers. A transcript must be translated, and the protein must reach the cell surface. Surface localization must be demonstrated in the relevant state, species and lesion stage. Its expression must be sufficiently selective to avoid harming protective VSMCs or other vascular cells. Finally, for therapeutic use, perturbing the molecule must change disease biology in a beneficial way. A molecule can satisfy one layer and fail the next [59]. CD200 and CD29/CD90/CD142 illustrate the value of surface markers for identification [33]. Fibroblast activation protein (FAP) illustrates how a state-enriched surface protein can become a depletion handle in preclinical therapy [16,60]. CD36 and triggering receptor expressed on myeloid cells 2 (TREM2) illustrate the danger of overextending macrophage or foam-cell biology into VSMC-selective targeting [61,62].

### 2.3. From Surface Marker to Therapeutic Target: A Translational Evidence Framework

For claim calibration, this review uses five translational evidence categories, which are each defined by a minimum evidentiary criterion. 1. State/lineage identification markers define VSMC origin or cellular state but do not require demonstrated surface accessibility. 2. Surface identification/sorting signatures require evidence supporting surface-protein expression and utility for live-cell identification or sorting but do not require evidence of therapeutic causality. 3. Functional surface interfaces require evidence supporting a surface-associated or membrane-localized role together with experimental evidence linking the molecule to VSMC phenotype, signaling, or plaque-relevant cellular function. 4. Causally supported candidate targets require perturbational evidence supporting a causal role in VSMC biology or plaque-relevant outcomes with cell- or lineage-restricted genetic evidence considered particularly informative; direct therapeutic targeting of the surface molecule is not required. 5. Intervention-supported surface targets require the direct targeting or exploitation of a surface-accessible molecule or surface-defined population in vivo with demonstrated plaque-relevant therapeutic effects.

Complementary human lesion-level evidence strengthens, but is not by itself sufficient for, assignment to this category.

In parallel with these evidence categories, we distinguish the translational role of a molecule or intervention. A surface marker identifies a cellular state or population but need not have a causal role in disease. A targeting handle is a surface-accessible molecule exploited for recognition or delivery without requiring that its direct perturbation alters VSMC biology. A causative therapeutic target is a molecule whose direct perturbation modifies VSMC phenotype or plaque-relevant outcomes. A therapeutic cargo is the delivered effector responsible for the intended biological reprogramming or therapeutic response.

**Figure 2:**
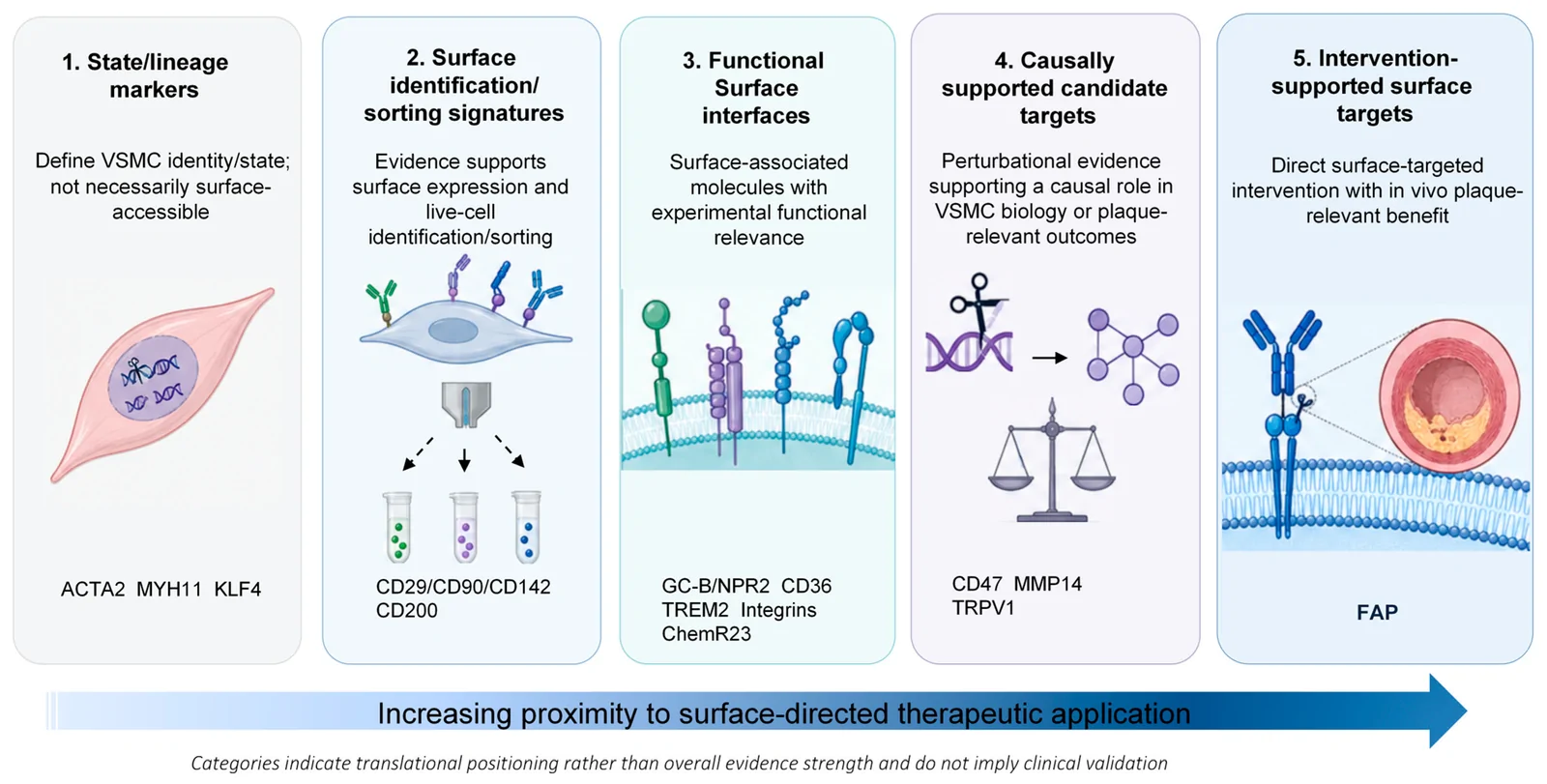
Translational evidence framework for VSMC surface markers and candidate targets in atherosclerosis. The arrow indicates increasing proximity to surface-directed therapeutic application; panel colors are used for visual distinction only.

Importantly, the 1–5 framework represents translational positioning rather than a strictly ordinal ranking of overall evidence strength. Progression from 1 to 5 reflects increasing proximity from state identification to surface-directed therapeutic application, but assignment to a later category does not necessarily indicate stronger evidence across every dimension. For example, cell-specific genetic perturbation may provide stronger evidence of VSMC-intrinsic causality than a systemic pharmacological intervention, whereas the latter may provide greater evidence of therapeutic tractability without establishing a VSMC-specific mechanism. Candidates should therefore also be evaluated across complementary dimensions, including surface localization, cellular/state specificity, mechanistic causality, human lesion validation, and direct intervention evidence. The progression from state/lineage identification to surface accessibility, functional relevance, causal support, and intervention-supported targeting is illustrated in Figure 2.

## 3. Cell-Surface Signatures for Identifying Modulated VSMCs

### 3.1. From Single-Cell Transcriptomics to Surface-Protein Validation

Single-cell transcriptomics provides the discovery layer for VSMC heterogeneity, but surface-protein validation is needed before transcriptional states can be isolated as live cells [16]. CITE-seq and related multimodal approaches address this gap by measuring transcriptomes and surface epitopes in the same cell [32,63]. In human carotid plaques, high-dimensional single-cell multimodal profiling linked transcriptional SMC/fibroblast-like states to surface proteins and supported the construction of flow cytometry panels for plaque-cell isolation [10,64]. In mouse lineage-tracing plaques, CITE-seq combined with rigorous VSMC ancestry assignment also identified surface markers that persist after VSMC modulation [13].

The significance of these studies is not merely technical. They show that VSMC states discovered by RNA can be translated into viable-cell workflows [65]. This changes what can be asked experimentally. Instead of inferring state from dissociated transcriptomes alone, investigators can isolate putative VSMC-derived populations, test their function, culture them, expose them to therapeutic agents or map their spatial distribution [64,65,66]. This is the first step toward precision intervention, but it remains an identification step [26]. It should not be confused with evidence that the same markers are therapeutic targets [67].

### 3.2. CD29–CD90–CD142: From Transcriptomic State to Live-Cell Sorting

The CD29/ITGB1-CD90/THY1-CD142/F3 combination is best positioned as a surface signature for live-cell identification and sorting [10]. Human plaque multimodal profiling used surface-protein information to distinguish SMC-derived and fibroblast-like populations [20], and the CD29/CD142/CD90 panel provided a practical route to isolate these cells [10]. CD90 has also been associated with non-contractile or modulated VSMC states in human lesions and lineage-traced mouse plaques, supporting its relevance to VSMC modulation [68]. CD142/F3 is a central component of tissue-factor–initiated coagulation, raising potential safety and specificity concerns for indiscriminate systemic targeting [69,70].

This distinction matters. The panel is valuable because it addresses a methodological bottleneck: how to recover modulated VSMC-derived cells after contractile markers have declined. It does not prove that CD29, CD90 or CD142 individually drive plaque progression. Nor does it establish that targeting these molecules would selectively remove pathogenic VSMCs. CD29/ITGB1 is a beta1-integrin subunit within extracellular-matrix adhesion signaling in VSMCs rather than a VSMC-specific therapeutic marker [71]; CD90 is not unique to VSMCs [72]; and CD142/F3 has coagulation-related functions that could create systemic risk if targeted indiscriminately [73]. The appropriate language is therefore “surface sorting signature” or “live-cell identification panel”—not “therapeutic target set”.

### 3.3. CD200: A Persistent Surface Lineage Marker for VSMC-Derived Cells

CD200 provides a complementary solution. Multimodal profiling identified CD200 as a cell-surface lineage marker of VSMCs and VSMC-derived cells in atherosclerosis [33]. The key value of CD200 is persistence [33]. Because modulated VSMC-derived cells may lose contractile markers, a surface marker that remains detectable across VSMC-derived populations can support lineage-informed identification and isolation [74].

CD200 is therefore most useful in the identification layer of the translational pipeline. It helps answer “which cells are VSMC-derived?” and supports live-cell workflows, particularly when combined with exclusion markers such as CD31 and CD45 [17,33]. Its VSMC-specific therapeutic relevance remains uncertain, because current evidence supports CD200 primarily as a marker of VSMC-derived plaque cells rather than as a causally validated regulator of plaque progression. Accordingly, CD200 is better regarded as a surface lineage/state marker than as a validated VSMC therapeutic target [75].

### 3.4. Combinatorial Signatures for VSMC State Separation

No single marker is likely to capture the full diversity of modulated VSMC states [76]. Atherosclerotic plaques contain cells with overlapping stromal, inflammatory, chondrogenic and lipid-loaded features [38,77]. Many surface molecules are shared across VSMCs, fibroblasts, macrophages, endothelial cells or immune cells [78]. Combinatorial signatures therefore provide a more realistic strategy than single-marker definitions. A panel can combine positive markers for VSMC-derived states, negative markers to exclude immune or endothelial lineages, and state-enriched proteins that distinguish biologically distinct or disease-relevant programs.

The move from single markers to combinations also reduces overinterpretation. CD90 alone may enrich for a modulated VSMC population in one context but does not uniquely define a single VSMC state across datasets. CD36 may reflect lipid uptake in both macrophages and VSMC-derived foam cells [79,80]. TREM2 strongly marks lipid-associated macrophage states and has also been implicated experimentally in SMC foam-cell formation, although its expression is not uniform across lineage-traced SMC-derived foamy populations [77,81,82]. A combinatorial panel could help determine whether these molecules occur on a VSMC-derived background, in a specific plaque region, and at a disease stage where intervention would be meaningful.

Combinatorial signatures also make it possible to separate technical identification from biological interpretation [78]. For example, a panel may be optimized to recover viable cells with high purity even if none of its markers is individually causal. This is a strength, not a weakness, when the goal is sorting [83]. The same panel may be inappropriate for therapy because each component has broad expression or essential functions outside the target state [84]. In practice, the first generation of VSMC surface signatures should be viewed as enabling technology: they permit the purification, culture, perturbation and spatial validation of cells that were previously accessible mainly through transcriptomic inference.

### 3.5. FAP: From Surface-State Identification to Therapeutic Actionability

FAP currently provides one of the clearest examples of progression from surface-state identification to preclinical therapeutic actionability. A recent human coronary artery disease atlas identified FAP-enriched modulated SMC populations within disease-associated neointimal plaque niches [16,85]. Unlike CD200 or CD29/CD90/CD142, whose current value is primarily identification or sorting, FAP provides a surface-accessible handle that has also been directly exploited in preclinical therapeutic intervention. Thus, FAP illustrates how a surface state marker can progress beyond identification when supported by intervention evidence [16,86].

However, although lineage-tracing studies indicate that plaque FAP-positive cells are predominantly derived from Myh11-positive VSMCs, the relative contribution of other FAP-expressing stromal populations to the therapeutic response cannot be completely excluded.

### 3.6. From Single Markers to Surface Identity

The main lesson from Section 3 is that surface identity is layered. CD29/CD90/CD142 and CD200 address the problem of identifying and isolating VSMC-derived cells after transcriptional reprogramming, whereas FAP provides preclinical proof-of-concept that a disease-associated surface state can also become therapeutically actionable. These examples should not be collapsed into a single category. Identification markers provide experimental access, whereas therapeutic targets require additional causal and intervention-level evidence. Together, these observations support a translational evidence framework for interpreting VSMC surface biology rather than a simple catalog of markers [87].

## 4. From Cell-Surface Identity to Selective Intervention and Plaque-Fate Modification

### 4.1. FAP: Selective Depletion of Pathogenic VSMC States

FAP-directed immunotherapy provides the best-developed preclinical example of selective depletion based on a VSMC-associated surface state [88]. Human plaque profiling identifies FAP-enriched modulated SMC populations, while FAP-directed bispecific T-cell engager (BiTE) therapy reduces atherosclerotic plaque burden in mouse models [16]. Together, these findings provide proof-of-concept that a surface-defined VSMC state can progress from identification to direct therapeutic intervention.

Importantly, this strategy should not be generalized to all modulated VSMCs. VSMC modulation is not uniformly detrimental, because some VSMC-derived populations contribute extracellular matrix, collagen deposition and fibrous-cap formation and may therefore support plaque stability [89,90]. The therapeutic objective is consequently the selective depletion of a disease-associated FAP-positive population rather than the broad suppression of VSMC phenotypic modulation.

FAP targeting nevertheless retains important translational limitations. FAP is not restricted to atherosclerotic VSMCs and is also expressed by activated fibroblastic and other stromal populations [91,92], raising potential off-target depletion and tissue-remodeling concerns [88,93]. Importantly, although lineage-tracing studies have provided evidence that FAP-positive plaque populations include Myh11-positive VSMC-derived cells, available intervention studies do not fully resolve whether the therapeutic benefit of FAP-directed depletion is specifically attributable to the elimination of VSMC-derived FAP-positive cells or also involves other FAP-expressing stromal populations. Thus, the depletion of non-VSMC FAP-positive stromal populations remains a potential off-target effect. Moreover, reduction in plaque burden alone does not establish improved plaque stability; the effects of FAP-directed intervention on collagen content, fibrous-cap integrity, lesion composition, and the preservation of non-target stromal populations and VSMC-derived populations potentially involved in matrix maintenance require further evaluation. FAP is therefore best regarded as an intervention-supported, preclinical surface target for disease-associated FAP-positive plaque populations rather than a definitively validated VSMC-specific therapeutic target.

### 4.2. Surface-Directed Delivery: CCR2-Targeted miR-145 as Reprogramming

C-C chemokine receptor type 2 (CCR2)-targeted microRNA-145 (miR-145) micelles represent a different therapeutic mode [94]. Here, the goal is not depletion but rather the delivery of a phenotypic reprogramming cargo. miR-145 is a central regulator of contractile VSMC identity, and reduced miR-145 is associated with VSMC modulation. Monocyte chemoattractant protein-1 (MCP-1) peptide-functionalized micelles were designed to exploit CCR2 expression on synthetic VSMCs to deliver miR-145, restore contractile features and suppress atherosclerotic progression in apolipoprotein E (ApoE)-deficient mice [19].

This strategy fits a “plastic/reversible state → modulate/reprogram” model, treating VSMC modulation as potentially reversible rather than terminally pathogenic. It also separates the surface handle from the therapeutic cargo: CCR2 provides cellular access, whereas miR-145 provides the reprogramming signal. This distinction is important because CCR2 is not VSMC-specific [95]. In addition to synthetic VSMCs, CCR2 is expressed by activated endothelial cells and myeloid populations, creating the potential for uptake outside the intended VSMC compartment [96]. The evidence therefore supports CCR2-targeted miR-145 delivery as a disease-directed reprogramming strategy but not as a VSMC-selective receptor therapy.

Accordingly, CCR2 should be interpreted as a targeting handle rather than a causative VSMC therapeutic target, whereas miR-145 represents the therapeutic cargo responsible for phenotypic reprogramming.

### 4.3. Functional and Causally Supported Surface Interfaces

#### 4.3.1. GC-B/NPR2 and the CNP/cGMP Axis

Guanylyl cyclase-B/natriuretic peptide receptor 2 (GC-B/NPR2) is a functional surface receptor that links extracellular C-type natriuretic peptide (CNP) to intracellular cyclic guanosine monophosphate (cGMP) signaling [97]. Single-cell analysis combined with live-cell cGMP imaging identified the C-type natriuretic peptide/guanylyl cyclase-B/cGMP (CNP/GC-B/cGMP) axis as a marker and regulator of modulated VSMCs in atherosclerosis. Importantly, this approach assessed receptor activity rather than expression alone. Modulated VSMCs showed altered natriuretic-peptide responsiveness, and SMC-specific loss of GC-B increased chondrocyte-like plaque-cell states, providing cell-specific causal evidence that GC-B signaling can influence VSMC phenotypic modulation [22].

The current evidence therefore supports GC-B/NPR2 primarily as a functional surface interface for state modulation, with emerging cell-specific causal support, rather than as an intervention-supported surface target. Human validation remains limited, particularly at the protein level, because reliable single-cell tissue detection of GC-B is technically challenging [22]. Moreover, natriuretic-peptide receptor expression and functional responsiveness can be altered by VSMC isolation and culture, emphasizing the importance of experimental context [98]. Although the pathway-level manipulation of CNP/cGMP signaling supports biological relevance [99], direct VSMC-specific GC-B intervention in human atherosclerosis remains unvalidated.

GC-B/NPR2 also illustrates a broader principle of surface-receptor biology: a receptor may be valuable because its activity reports and regulates a reversible cellular state rather than because it uniquely identifies a lineage. In this setting, the therapeutic concept is the modulation of receptor signaling rather than the depletion of receptor-positive cells. Functional assays are therefore particularly important [100], because receptor transcript abundance at a single time point may not accurately reflect ligand responsiveness or downstream signaling capacity [101].

#### 4.3.2. MMP14 as a Membrane-Associated Regulator of SMC State

Matrix metalloproteinase 14 (MMP14), also known as MT1-MMP, is a membrane-type metalloproteinase that can regulate extracellular matrix remodeling, migration and signaling [102]. Adult SMC-specific Mmp14 deletion in Ldlr-deficient mice reduced atherosclerosis progression, enhanced regression and shifted SMC populations away from foam-cell-like states, while human coronary fibroatheromas showed increased SMC-associated MMP14 expression [24]. Additional vascular evidence shows that MMP14 regulates VSMC signaling through LRP6 shedding, linking its cell-surface proteolytic activity to proliferative and migratory responses [103]. Together, these findings provide strong support for MMP14 as a VSMC-relevant membrane-associated interface with cell-specific causal evidence in atherosclerosis.

MMP14 therefore reaches the level of a causally supported candidate target. It has human plaque protein relevance, adult SMC-specific genetic evidence and plaque outcome data. The principal unresolved issues are pharmacological selectivity and therapeutic window. Historically, broad-spectrum MMP inhibitors showed limited clinical benefit and dose-limiting musculoskeletal toxicity, underscoring the need for target-selective inhibition rather than implicating MMP14 specifically [104]. MMP14 also functions in multiple cell types and tissue-remodeling contexts, creating an additional requirement for cell- and context-selective intervention [102]. Accordingly, MMP14 is best positioned as a high-priority causally supported candidate target for modulating disease-associated SMC state transitions rather than as a clinically validated surface-directed therapy.

#### 4.3.3. ChemR23 as a Context-Dependent VSMC-Preserving Receptor

Chemerin chemokine-like receptor 1 (ChemR23/CMKLR1) illustrates why the cellular compartment matters in surface-receptor biology [105]. A recent VSMC-focused study showed that non-hematopoietic ChemR23 deficiency increased lesion size, lesional VSMC proliferation and VSMC foam-cell accumulation, while human plaque analyses showed higher CMKLR1 expression in contractile VSMCs. In cultured human aortic SMCs, ChemR23 inhibition promoted synthetic and macrophage-like features, increased proliferation and cholesterol uptake, whereas agonist signaling counteracted several of these responses [67].

These findings support ChemR23 as a context-dependent functional receptor that may help preserve contractile VSMC identity and restrain foam-cell-like switching [106]. However, ChemR23 also functions in immune compartments, particularly in macrophages and other myeloid cells, and its biological effects depend on both cellular context and ligand environment [107,108]. Systemic targeting could therefore act through several cell populations rather than VSMCs alone [105]. ChemR23 is therefore best classified as a functional surface interface with compartment-level causal support and candidate therapeutic potential rather than as a causally validated VSMC-specific therapeutic target.

#### 4.3.4. CD47: A VSMC-Relevant Surface Checkpoint

CD47 functions as a “do not eat me” signal that regulates efferocytosis through interactions with signal regulatory protein alpha (SIRPα) on phagocytes. In atherosclerosis, systemic CD47 blockade has been linked to improved efferocytosis and plaque benefit, but that evidence alone does not prove VSMC-specific action [109]. Newer work strengthens the VSMC case: SMC-specific Cd47 deletion suppresses atherosclerosis, reduces macrophage burden and necrotic area, and increases the efferocytosis of apoptotic VSMCs, while CD47 knockdown in human VSMCs counteracts thrombospondin-1-induced dedifferentiation [15]. Together, these findings support a model in which VSMC CD47 limits the clearance of apoptotic VSMCs and may thereby contribute to necrotic-core expansion and plaque-destabilizing features [110].

CD47 can therefore be classified as a causally supported candidate target with a VSMC-relevant surface-checkpoint function. The appropriate therapeutic claim remains bounded. Genetic deletion in SMCs supports causality, and human VSMC experiments support cell relevance [111]. Yet systemic CD47 blockade affects multiple cell types and may cause on-target hematologic toxicity [112]. Within a VSMC-directed framework, CD47 therefore remains better classified as a causally supported candidate target rather than an intervention-supported VSMC surface target of the type represented by FAP.

The CD47 example is useful because it separates three related but distinct therapeutic ideas. One idea is to block CD47 systemically to restore phagocytosis within plaques [12,110]. A second is to reduce CD47 signaling within VSMCs to limit maladaptive phenotypic change and improve the clearance of apoptotic VSMCs. A third is to deliver a VSMC-selective CD47 intervention only to a pathological surface-defined state. The first has broader anti-CD47 precedent, the second is supported by emerging VSMC-specific genetic evidence, whereas the third remains largely conceptual. These intervention levels should not be conflated when evaluating the translational maturity of VSMC-selective CD47 targeting [113,114].

#### 4.3.5. CD36-TREM2: Lipid Uptake, Foam-Cell States and Lineage Ambiguity

CD36 is a scavenger receptor that mediates modified-lipoprotein uptake. VSMC-derived foam cells contribute to the lipid-laden plaque-cell compartment, and recent studies directly implicate CD36 expression and membrane trafficking in VSMC foam-cell formation [18,74]. Older human work identified a smooth-muscle-associated CD36 transcript variant in cultured human VSMCs and atherosclerotic plaques, and antisense inhibition reduced oxidized low-density lipoprotein (oxLDL) uptake [115]. More recent work linked Axin interactor, dorsalization associated (AIDA) to CD36 membrane translocation, VSMC-derived foam-cell formation and plaque burden [18]. Taken together, these findings support CD36 as a functional lipid-uptake interface in VSMCs.

TREM2 requires greater caution. Importantly, a foam-cell phenotype should not be equated with lineage identity; lipid loading or the expression of macrophage-associated genes does not by itself establish VSMC origin [10]. TREM2 promotes cholesterol uptake and foam-cell formation through mechanisms that include CD36 regulation with experimental evidence involving both macrophages and SMCs [81]. This creates a mechanistic bridge between TREM2 and CD36 but does not establish TREM2 as a VSMC-selective target. TREM2-positive foam-cell populations are prominent within the myeloid compartment of human atherosclerotic plaques [10,116]. Accordingly, TREM2 is best regarded as a macrophage-overlapping foam-cell interface, while current evidence is insufficient to support VSMC-selective therapeutic targeting.

#### 4.3.6. TRPV1 and Channel-Based Intervention

Transient receptor potential vanilloid 1 (TRPV1) is a surface ion channel that has been explored as an intervention-accessible pathway in vascular disease. Recent studies further support a functional role for TRPV1 in regulating VSMC proliferation, migration and phenotypic behavior [117]. Copper sulfide nanoparticles acting as a photothermal switch for TRPV1 signaling attenuated atherosclerosis in preclinical models [118]. More recent genetic evidence also indicates that the loss of TRPV1 aggravates experimental atherosclerosis and plaque instability, although these effects are not VSMC-specific [119].

The evidence is intriguing but remains preclinical. TRPV1 lacks strong human plaque validation as a VSMC-state-specific surface target, and nanoparticle-based photothermal delivery introduces additional translational barriers [120].

#### 4.3.7. Integrins: Adhesion Interfaces Rather than Single Targets

Integrins are central to VSMC adhesion, migration, matrix sensing and phenotype regulation. They are natural surface interfaces because they connect extracellular matrix composition to intracellular signaling [71]. Recent studies further show that fibronectin-dependent integrin signaling can regulate VSMC phenotypic and inflammatory programs in atherosclerotic lesions, while β1- and αVβ3-containing integrins link matrix remodeling to focal adhesion kinase (FAK), phosphoinositide 3-kinase (PI3K) and related signaling pathways [121,122]. However, integrins as a broad class are too heterogeneous and widely distributed to be treated as a single precision target.

Integrins should therefore be interpreted as heterodimer- and context-dependent functional surface interfaces whose therapeutic relevance depends on the specific heterodimer, VSMC state, lesion compartment and delivery mode. Table 2 summarizes the translational evidence categories, functional roles, and major evidence boundaries of the representative surface markers and candidate targets discussed above.

### 4.4. Plaque Burden, Lesion Composition and Fibrous-Cap Integrity

Surface-directed VSMC intervention should not be judged only by plaque size. Some interventions may reduce lesion area by suppressing foam-cell formation or inflammatory expansion. Others may alter cellular composition without dramatically changing total plaque burden. FAP-directed depletion, MMP14 deletion, CCR2-miR-145 delivery, TRPV1 activation and ChemR23 modulation all affect plaque biology through different routes. The more meaningful question is whether these changes shift plaques toward a composition associated with greater stability [40].

For pathogenic states, depletion may reduce harmful stromal-inflammatory programs. For plastic states, reprogramming may restore contractile or matrix-supportive functions. For protective states, preservation may be more important than suppression [89]. This is why a surface target’s effect on plaque composition must be interpreted together with its effect on fibrous cap structure, lipid burden, inflammation, necrosis and calcification [123].

VSMCs are central to fibrous cap formation and collagen production [124]. A therapy that reduces VSMC-derived pathogenic cells but also removes cap-supporting cells could increase risk even if plaque area declines [125]. MMP14 and FAP illustrate the importance of this balance. Adult SMC-specific MMP14 deletion reduced plaque progression, enhanced regression and shifted SMC composition toward fibroblast-like and away from foam-cell-like states, suggesting that disease-promoting SMC programs can be restrained without indiscriminate loss of the SMC compartment [24]. FAP-directed depletion requires careful interpretation because FAP-positive cells may include activated fibroblastic and other stromal populations involved in matrix remodeling.

For FAP-directed depletion in particular, reduced plaque burden should not be interpreted as evidence of improved plaque stability in isolation [16]. Therapeutic evaluation should also consider collagen content, fibrous-cap thickness and integrity, necrotic-core characteristics, and the overall cellular composition of the residual plaque. Whether these plaque features are preserved or improved after FAP-directed intervention requires further investigation.

The cap-focused endpoint is therefore essential. A precision VSMC therapy should not simply reduce VSMC number. It should reduce the harmful state while preserving or restoring matrix-producing, cap-stabilizing programs [126].

This requirement has practical consequences for preclinical study design. Interventions should report not only the en face lesion area or aortic root plaque size but also cap thickness, collagen content, SMC-derived matrix contribution, necrotic core area, macrophage burden, efferocytosis and calcification pattern [127]. When possible, these endpoints should be linked back to lineage-resolved VSMC states. A therapy that reduces foam-cell-like VSMCs while expanding fibromyocyte-like or collagen-producing states would support the preserve/reprogram logic. A therapy that reduces both harmful and protective VSMC-derived compartments would need a much narrower therapeutic window.

### 4.5. Inflammation, Lipid Handling and Calcification

Modulated VSMCs interact with immune cells through cytokines, chemokines, lipid handling, matrix remodeling and efferocytosis [128]. CD47 sits at the interface between apoptotic VSMCs and macrophage clearance [15]. CD36 and TREM2 connect lipid uptake to foam-cell biology [81]. ChemR23 links vascular cell state to inflammatory resolution pathways [67]. These interfaces show that VSMC targeting can reshape the plaque microenvironment without directly targeting immune cells.

Yet this same interdependence creates attribution problems [129]. If a therapy reduces macrophage burden after manipulating a VSMC surface molecule, the effect may be VSMC-intrinsic, immune-mediated or both. Mechanistic attribution should therefore follow the experimental design: SMC-specific genetic perturbation provides stronger evidence of VSMC-intrinsic causality than systemic ligand or pharmacological treatment [130]. Human plaque association supports relevance but not mechanism.

VSMC-derived chondromyocyte-like and osteogenic states contribute to calcification and plaque remodeling. GC-B/NPR2 is particularly relevant here because the CNP/GC-B/cGMP axis is linked to chondrocyte-like VSMC states [22]. Modulating this pathway may influence calcification-associated state transitions, but the evidence remains largely mechanistic and preclinical.

Calcification also illustrates why state labels cannot be assumed to be uniformly harmful. Microcalcification, macrocalcification and chondrogenic programs may have different implications depending on plaque stage and spatial pattern [131]. Surface-directed therapies that affect chondromyocyte-like states should therefore be evaluated by spatial and mechanical plaque outcomes—not only by marker changes.

### 4.6. Integrated Therapeutic Benefit and State-Matched Plaque Outcomes

The final measure of surface-directed VSMC targeting should be integrated plaque benefit. That includes reduced pathogenic cell burden, preserved fibrous cap integrity, lower inflammatory amplification, controlled lipid accumulation, reduced necrotic core expansion and favorable calcification pattern [127,132].

This integrated standard is more demanding than lesion area, but it is necessary for precision intervention [133]. A target that reduces plaque size while weakening the cap would not be clinically attractive. Conversely, a target that modestly changes plaque area but strongly improves composition could be valuable [134]. These state-matched therapeutic modes and their intended plaque-fate outcomes are summarized in Table 3.

## 5. Challenges and Future Perspectives

### 5.1. Surface Specificity, Therapeutic Tractability and Evidence Boundaries

Therapeutic suitability depends on more than surface expression and requires a consideration of accessibility, state specificity, functional relevance and druggability [59]. Most candidate molecules are not unique to VSMCs. CD36, TREM2, CD47, integrins, CCR2 and FAP are also expressed in other cell types or disease contexts. Even when a marker is enriched in a VSMC-derived state, enrichment does not imply exclusivity [137]. Precision targeting will therefore likely require combinatorial recognition, spatially restricted delivery, disease-activated payloads or temporally defined treatment windows [138,139].

Evidence must also be interpreted across molecular and experimental levels. Transcript enrichment does not establish protein abundance or cell-surface localization; surface localization does not establish VSMC-state specificity; and systemic intervention does not establish a VSMC-intrinsic mechanism [140]. Conversely, cell-specific genetic perturbation can demonstrate causality without establishing pharmacological selectivity or druggability [141]. Candidate surface targets should therefore be evaluated across complementary dimensions including molecular localization, cellular and state specificity, human lesion relevance and direct intervention evidence [142].

Druggability represents an additional barrier. A surface marker can be accessible but biologically inert. CD200 and CD29/CD90/CD142 are useful for identification or sorting, but current evidence does not establish them as VSMC-selective therapeutic targets [33]. Conversely, MMP14, ChemR23 and GC-B/NPR2 have functional evidence, but therapeutic exploitation requires the careful control of systemic and off-target effects [143].

### 5.2. Harmful Versus Protective Modulated VSMC States

**Figure 3:**
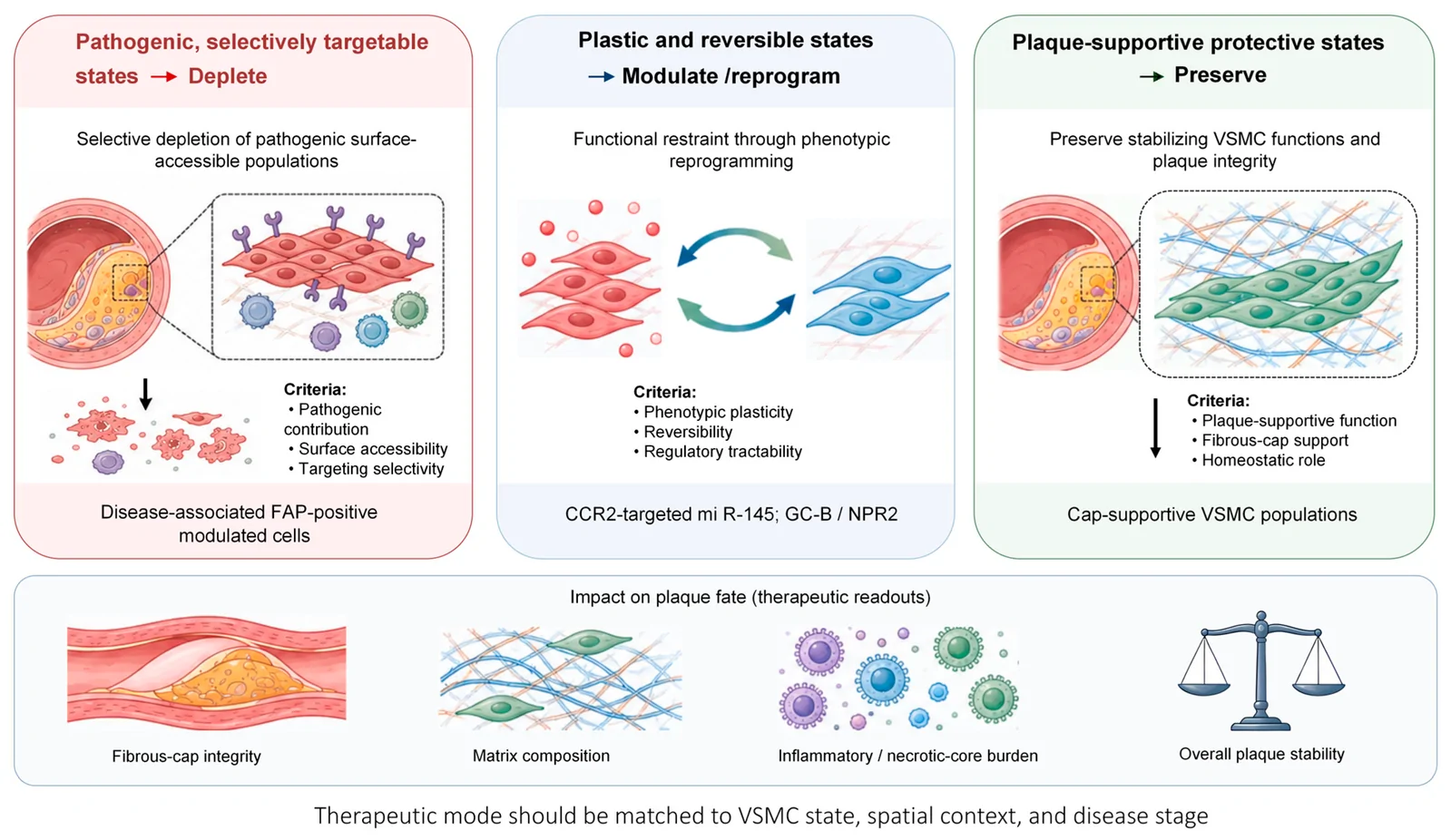
State-guided therapeutic modes for VSMC intervention in atherosclerosis. Red, blue, and green denote depletion, modulation/reprogramming, and preservation strategies, respectively; arrows indicate intervention flow or phenotypic transitions.

The field must avoid treating VSMC modulation as a binary loss of identity. Modulated VSMCs can exert pathogenic, reversible or plaque-stabilizing functions depending on their cellular program, spatial location and disease stage [8,144]. We therefore propose that the deplete–modulate/reprogram–preserve framework should be viewed as a state-dependent decision model rather than a fixed classification of individual targets. Depletion may be appropriate when a surface-accessible state has evidence of pathogenic activity and can be removed without compromising cap-supportive VSMCs [145]. Reprogramming may be preferable when a pathological state remains biologically plastic and potentially reversible [95], whereas preservation may be prioritized for matrix-producing populations that contribute to fibrous-cap integrity [146]. Assignment to these therapeutic modes should consider pathogenic contribution, surface accessibility, targeting selectivity, phenotypic plasticity, reversibility, regulatory tractability, spatial localization, and disease stage [51]. FAP-positive modulated SMCs currently provide the clearest example of depletion-oriented intervention [147,148], whereas CCR2-directed miR-145 delivery illustrates a reprogramming strategy [135] and GC-B/NPR2 represents a functional pathway with potential for state modulation [22]. This state-dependent therapeutic decision framework is illustrated in Figure 3.

### 5.3. Species, Spatial and Disease-Stage Heterogeneity

Mouse models are essential but incomplete. Mouse lineage tracing provides causal ancestry that cannot be replicated easily in humans, but human plaques differ in size, age, hemodynamics, treatment exposure and rupture biology [37]. Human single-cell and spatial atlases improve relevance but often lack causal perturbation [149]. The strongest translational evidence will therefore require convergence across lineage-defined causality, human state mapping, protein-level surface localization, spatial validation and direct intervention [150].

Spatial context is equally important. The same VSMC-derived state may have different meaning in the fibrous cap, shoulder region, necrotic core border, media or adventitia. A surface target that appears attractive in dissociated cells may be inaccessible, non-specific or functionally different in tissue [151].

Human validation should therefore be multi-layered. A convincing human claim should ideally include transcript detection, protein detection, spatial localization, relationship to VSMC-derived or SMC-like identity, and association with plaque features relevant to stability [39,149,152]. For therapeutic claims, human data alone will rarely prove causality, but they can prevent a mouse-specific target from being overpromoted [37]. Conversely, mouse lineage tracing can prove ancestry and mechanism but cannot substitute for human lesion relevance [87].

### 5.4. Build Spatially Resolved VSMC Surface Atlases

The next phase should build unbiased, spatially resolved surface atlases of VSMC-derived plaque states. These atlases should combine lineage tracing, CITE-seq, spatial transcriptomics, multiplexed protein imaging and functional validation [153,154]. The goal is not only to name markers but rather to identify surface combinations that distinguish pathogenic, plastic and protective states.

Surface signatures should be interpreted together with spatial information [64]. A marker’s meaning may depend on whether it is expressed in the fibrous cap, necrotic-core border, shoulder region or media [154,155]. Spatially informed targeting may therefore reduce off-target effects by combining molecular identity with lesion location and disease-stage context [156,157].

### 5.5. State-Matched Intervention, Therapeutic Stratification and Theranostics

A major future goal is to move from individual surface-targeted interventions toward state-matched therapeutic strategies. Rather than assigning a fixed therapeutic meaning to individual targets, future studies should determine which molecular and spatial signatures identify lesions most likely to benefit from depletion, reprogramming, functional modulation or preservation [158]. Combination strategies may also become relevant when multiple VSMC states coexist within the same plaque [154].

A longer-term goal is to determine whether lesion-level surface signatures can support state-informed therapeutic stratification. FAP-enriched disease-associated stromal states may ultimately favor depletion-oriented approaches, whereas lesions enriched in lipid-loaded VSMC states may be more amenable to lipid-handling or state-modulating interventions [159,160]. However, such stratification remains a research framework rather than a clinically actionable algorithm. Prospective studies must establish whether surface signatures reproducibly quantify VSMC-state burden, predict plaque behavior and identify response to state-matched interventions.

Surface targets also create opportunities for molecular imaging and theranostics. FAP imaging already suggests that surface-accessible plaque biology can be visualized in human disease [161,162]. Future imaging strategies could help identify lesions enriched for particular surface-defined states and link molecular imaging phenotypes to state-matched interventions. Such approaches will require careful validation that imaging signals reflect the intended VSMC-derived state rather than broader stromal or inflammatory compartments [163].

### 5.6. Concluding Perspective

The translational promise of VSMC single-cell biology depends on moving beyond transcriptional state definitions toward surface-accessible, functionally meaningful and therapeutically calibrated targets. Current evidence shows that surface signatures can enable the live-cell identification of modulated VSMC-derived populations, whereas only a smaller subset of surface molecules has sufficient causal or intervention evidence to support therapeutic consideration [10,33]. Importantly, VSMC modulation should not be treated as a uniformly harmful process: pathogenic states may warrant depletion, plastic states may be candidates for modulation or reprogramming, and matrix-supportive states may warrant preservation [16,20,160]. Future progress will therefore depend on integrating lineage, surface-protein, spatial, functional and intervention evidence to determine not simply whether VSMCs change state but also which states can be safely identified, accessed and manipulated to improve plaque stability.

## Figures and Tables

**Table 1:** Representative disease-associated VSMC states reported across species, vascular contexts, and disease settings.

Species/Experimental Context	State Designation	Evidence Basis	Representative Markers	Spatial Context and Functional Interpretation
Human coronary artery	Fibromyocyte-like state	scRNA-seq and spatial profiling (state inference)	VCAN, COL1A1, LTBP2, TNFRSF11B [42]	Neo-intima; associated with ECM remodeling and fibrotic transition
Mouse atherosclerosis models	Fibromyocyte-like state associated with VSMC lineage	Lineage tracing and transcriptomic profiling	VCAN, COL1A1-related ECM genes	Neo-intima; ECM remodeling and matrix deposition
Human coronary artery	Chondromyocyte-like state	scRNA-seq and spatial profiling	CCL19, COMP, POSTN, MGP [43]	Neo-intima; chondrogenic-like matrix remodeling
Mouse atherosclerosis models	Chondrogenic-like state associated with VSMC lineages	Lineage-associated studies and transcriptomic profiling	ECM/chondrogenic-related genes [43,44]	Lesional intima; matrix remodeling
Mouse plaque models	VSMC-lineage-derived foam-cell-like state	Myh11 lineage tracing and transcriptomics	LGALS3, CD68, LPL, FABP4 [7,45]	Lipid-rich plaque regions; lipid accumulation and inflammatory remodeling
Human atherosclerotic plaque datasets	Foam-cell-like state with inferred VSMC contribution	Transcriptomic and spatial inference	LGALS3, CD68, LPL-related signatures [9,39]	Plaque regions; inflammatory/lipid-associated remodeling

Note: Disease-associated VSMC states represent context-dependent cellular states rather than fixed cell types. Similar state labels across species indicate shared molecular or functional features rather than identical cellular identities. Lineage tracing provides direct ancestry evidence in experimental models, whereas human plaque states are inferred mainly from transcriptomic, proteomic, and spatial approaches.

**Table 2:** Translational evidence categories of representative VSMC surface markers and candidate targets in atherosclerosis.

Candidate	Translational Evidence Category	Evidence Profile	Main Use	Evidence Boundary
CD29/ITGB1-CD90/THY1-CD142/F3	2—Surface identification/sorting signature	RNA + protein + surface validation; human state association	Live-cell identification and sorting	Useful as a combinatorial surface signature, but direct therapeutic causality has not been demonstrated [10]
CD200	2—Surface identification/sorting signature	RNA + protein + lineage/state identification; limited intervention	Persistent VSMC lineage/state identification	Supports lineage-aware live-cell identification; no direct intervention evidence [33]
FAP	5—Intervention-supported surface target	Surface + spatial + lineage + intervention evidence	Pathogenic-state depletion	Preclinical FAP-targeted efficacy is supported, but FAP is not VSMC-specific and lineage attribution of the therapeutic benefit remains unresolved [16]
CCR2-miR-145	Targeting strategy (not a causal-target category): CCR2 as targeting handle; miR-145 as therapeutic cargo	Targeting delivery evidence; cargo-mediated intervention	Targeted delivery and phenotypic reprogramming	Preclinical targeted-delivery efficacy is supported, but CCR2 is not VSMC-specific and functions primarily as a targeting handle; the phenotypic reprogramming effect is mediated by the miR-145 cargo [19,94]
GC-B/NPR2	3—Functional surface interface	Functional receptor evidence; limited human surface validation	Functional receptor/state modulation	Functional regulation of modulated VSMC states is supported, but human protein-level and surface validation remain limited [22]
CD47	4—Causally supported candidate target	Surface expression + genetic causal evidence; limited intervention	Surface checkpoint/efferocytosis interface	SMC-specific genetic evidence supports causality, but a VSMC-selective pharmacological strategy has not been validated [15]
CD36	3—Functional surface interface	RNA/protein + lipid uptake function; limited VSMC specificity	Lipid uptake and membrane trafficking	Supports VSMC lipid-uptake biology, but expression and foam-cell functions are not VSMC-specific [18,74]
TREM2	3—Functional surface interface	RNA/protein + foam-cell interface; macrophage overlap	Macrophage-overlapping foam–cell interface	Strong myeloid overlap limits lineage specificity; current evidence is insufficient for VSMC-selective therapeutic targeting [62,81]
Integrins	3—Functional surface interface	Surface biology + functional signaling; context dependent	Adhesion, migration and matrix sensing	Therapeutic interpretation depends on the specific heterodimer, VSMC state, lesion compartment and delivery mode [121]
MMP14/MT1-MMP	4—Causally supported candidate target	Surface + genetic causal evidence; limited therapeutic targeting	Membrane-associated state regulation	Adult SMC-specific genetic evidence supports causality, but selective pharmacological druggability remains unresolved [24]
TRPV1	4—Causally supported candidate target	Surface + functional intervention evidence; human validation limited	Channel-based intervention	Direct preclinical intervention evidence exists, but human plaque validation and VSMC-state specificity remain limited [118,119]
ChemR23/CMKLR1	3-Functional surface interface	Functional receptor evidence; compartment-level causal support	Context-dependent VSMC preservation/state modulation	Cultured SMC studies support direct functional relevance, while non-hematopoietic deletion provides compartment-level in vivo causal support; however, definitive SMC-specific in vivo genetic validation is lacking [67]

Note: Categories indicate translational positioning rather than a strictly ordinal ranking of overall evidence strength. Assignment is based on the minimum defining criteria for each category and does not imply superiority across complementary evidence dimensions or clinical validation. Evidence profiles summarize the major evidence dimensions supporting each candidate, including molecular expression, surface accessibility, spatial validation, human lesion evidence, and intervention data.

**Table 3:** Deplete–modulate/reprogram–preserve framework.

VSMC State Logic	Therapeutic Mode	Candidate Examples	Plaque-Fate Goal
Pathogenic, surface-accessible state	Deplete	FAP-positive modulated SMCs [16]	Reduce harmful stromal/inflammatory burden while monitoring fibrous-cap integrity
Plastic or reversible state	Modulate/Reprogram	CCR2-miR-145 [135]; GC-B/NPR2 [22]	Shift VSMC states toward less pathogenic or more stabilizing programs
Lipid-loaded foam-cell-like state	Limit uptake or trafficking	CD36; TREM2-CD36 axis [81]; AIDA-CD36 trafficking [18]	Reduce foam-cell burden without assuming macrophage specificity
Matrix-degrading or migratory state	Functional inhibition	MMP14 [24]; selected integrins [136]	Reduce progression while preserving cap-supporting SMC functions
Matrix-producing, cap-supportive VSMC-derived state	Preserve	Collagen-producing VSMC-derived/fibromyocyte-like populations [8,40]	Maintain extracellular-matrix production, fibrous-cap integrity and plaque stability

Note: Assignment to depletion, modulation/reprogramming or preservation is state- and context-dependent rather than an intrinsic property of an individual target. Therapeutic mode should consider pathogenic causality, state reversibility, surface accessibility and selectivity, spatial localization, disease stage and the potential loss of plaque-stabilizing VSMC functions.

## Data Availability

No new data were created or analyzed in this paper. Data sharing is not applicable to this article.

## Abbreviations

**Abbreviation**	**Definition**
VSMC	vascular smooth muscle cell
SMC	smooth muscle cell
ECM	extracellular matrix
MMPs	matrix metalloproteinases
TGF-β1	transforming growth factor-β1
CITE-seq	cellular indexing of transcriptomes and epitopes by sequencing
scRNA-seq	single-cell RNA sequencing
FACS	fluorescence-activated cell sorting
FAP	fibroblast activation protein
CD29/ITGB1	integrin β 1
CD90/THY1	Thy-1 cell surface antigen
CD36	CD36 molecule (fatty acid translocase)
CD47	CD47 molecule (integrin-associated protein)
CD200	CD200 molecule
CD142/F3	tissue factor (coagulation factor III)
THY1/CD90	Thy-1 cell surface antigen
CCR2	C-C chemokine receptor type 2
MCP-1	monocyte chemoattractant protein-1
miR-145	microRNA-145
NPR2	natriuretic peptide receptor 2
GC-B	guanylyl cyclase-B
cGMP	cyclic guanosine monophosphate
CNP	C-type natriuretic peptide
CNP/GC-B/cGMP axis	C-type natriuretic peptide/guanylyl cyclase-B/cyclic guanosine monophosphate signaling axis
MYH11	myosin heavy chain 11
TAGLN	transgelin
CNN1	calponin 1
KLF4	Kruppel-like factor 4
MMP14	matrix metalloproteinase 14
ChemR23	chemerin receptor 23
SIRPα	signal regulatory protein alpha
TREM2	triggering receptor expressed on myeloid cells 2
oxLDL	oxidized low-density lipoprotein
AIDA	Axin interactor, dorsalization associated
TRPV1	transient receptor potential vanilloid 1
FAK	focal adhesion kinase
PI3K	phosphoinositide 3-kinase
